# The Influence of Treadmill Training on the Bioelectrical Activity of the Lower Limb Muscles in Patients with Intermittent Claudication

**DOI:** 10.3390/jcm11051302

**Published:** 2022-02-27

**Authors:** Anna Mika, Piotr Mika, Łukasz Oleksy, Anita Kulik

**Affiliations:** 1 Institute of Clinical Rehabilitation, University of Physical Education in Kraków, 31-571 Krakow, Poland; piotr.mika@awf.krakow.pl; 2 Orthopaedic and Rehabilitation Department, Medical University of Warsaw, 02-091 Warsaw, Poland; loleksy@oleksy-fizjoterapia.pl; 3 Physiotherapy and Sports Centre, Rzeszow University of Technology, 35-959 Rzeszow, Poland; 4 Oleksy Medical & Sports Sciences, 37-100 Lancut, Poland; 5 Department of Physiotherapy, Faculty of Physical Culture in Gorzów Wielkopolski, Poznań University of Physical Education, 61-871 Poznan, Poland; konikanita@gmail.com

**Keywords:** intermittent claudication, muscles, electromyography, sEMG

## Abstract

Aim: Intermittent claudication is the most common symptomatic manifestation of peripheral arterial disease (PAD), presenting as ischemic leg muscle pain and gait dysfunction. The aim of this study was to evaluate the changes in bioelectrical activity of the lower limb muscles activity in claudicating patients over a 12-week period of supervised treadmill training and to verify the hypothesis as to which muscles of lower limbs are activated by training treatment—the proximal, as compensatory mechanism, or the distal, which are the most ischemic. Methods: The study comprised 45 patients aged 60–70 years (height 168.8 ± 6.8 cm, weight 78.9 ± 9.2 kg) with PAD and unilateral intermittent claudication (Fontaine stage IIa/IIb), who participated in a 12-week supervised treadmill training program. Surface electromyography (sEMG) of the gastrocnemius lateralis (GaL), gastrocnemius medialis (GaM), tibialis anterior (TA), biceps femoris (BF), rectus femoris (RF) and gluteus medius (GM) muscles in the claudicated leg were continuously measured during the treadmill test. The average mean amplitude and mean amplitude range of the sEMG signal were analyzed. Results: During the treadmill test, after 12 weeks of training, the average mean amplitude of the GM (105 ± 43 vs. 74 ± 38%, *p* = 0.000008, ES = 0.76), BF (41 ± 22 vs. 33 ± 12%, *p* = 0.006, ES = 0.45) and GaM (134 ± 50 vs. 114 ± 30%, *p* = 0.007, ES = 0.48) muscles was significantly lower compared with baseline. The mean amplitude range was significantly decreased after 12 weeks of training in the GM (229 ± 64 vs. 181 ± 62%, *p* = 0.008, ES = 0.77) and BF (110 ± 69 vs. 84 ± 31%, *p* = 0.0002, ES = 0.48) muscles. After 12 weeks of training, the mean amplitude range of the TA muscle was significantly higher compared with baseline (104 ± 46 vs. 131 ± 53%, *p* = 0.001, ES = 0.54), but without significant changes in the average mean amplitude value. The most favorable changes, suggesting the lowest muscle fatigue and the highest walking capacity, were found in patients with the longest walking time. Conclusions: The obtained results may suggest that after 12 weeks of treadmill training, beneficial changes occurred in both the proximal and distal muscles. Therefore, greater foot plantar flexion and stronger push-off as well as greater hip extension may be considered the main mechanisms of observed gait pattern improvement. It may also be suggested that the therapy of gait alterations in patients with PAD should be focused not only on calf muscle pump improvement, but also on proximal hip extensor strengthening.

## 1. Introduction

Intermittent claudication is the most common symptomatic manifestation of peripheral arterial disease (PAD), presenting as ischemic leg muscle pain and gait dysfunction [1]. Claudication pain is the result of walking induced reduction in blood supply to the working muscle of the lower extremity. Intermittent claudication and its associated ambulatory dysfunction are associated with poor health outcomes, physical dependence and impaired quality of life [2,3].

The nature of PAD gait is overall “sluggish and tired”. Patients with claudication have decreased gait velocity, stride and step length, while, at the same time, increased step width, which favors greater gait stability [1,4,5]. Based on kinematic and kinetic data, Chen et al. [6] have observed a weakness in the posterior muscles of the hip and calf, suggesting it as the key factor underlying alterations in PAD gait. Additionally, significant deficits in ankle plantar flexor strength was noted [7,8,9,10]. Moreover, it was reported that the gait pattern is abnormal with the first steps taken, even in the absence of pain, and worsens after the onset of claudication pain [4,11,12].

The mechanism of exercise-induced improvement in walking ability is not established. However, limited and equivocal evidence has been found in the literature regarding the effects of exercise training on leg-muscle performance in patients with claudication. As was suggested by TASC guidelines, possible explanations for improvement in walking ability may be improved metabolic capacity in the muscles and altered gait using the more proximal non-ischemic muscle, by analogy to amputees walking with a prosthesis [13]. In contrast, Wang et al. [14] have suggested that supervised treadmill training can result in an improved calf muscle contractile function, and the change in walking capacity is correlated with the change in calf muscle endurance.

It has been reported that during fatiguing muscle contractions, changes in the sEMG signal have been reported as an increase in the amplitude and a decrease in the frequency content [15,16]. In prolonged muscle contractions, the amplitude of sEMG increases in relation to the increasing number of newly recruited motor units, which replace those previously active [16,17]. In some studies, bioelectrical activity of the lower limb muscles has been compared between PAD patients and healthy controls [11,18]. To our knowledge, there is no existing study in which changes in bioelectrical activity of the lower limb muscles, due to implementing a walking training program in PAD patients, would be examined using surface electromyography (sEMG). Only in our previous pilot study was sEMG of the leg muscles in claudicating patients measured during treadmill walking before and after 12 weeks of training. We observed improvement in gastrocnemius lateralis muscle bioelectrical activity [19].

The aim of this study was to evaluate changes in the bioelectrical activity of the lower limb muscles in claudicating patients over 12-weeks of supervised treadmill training and to verify the hypothesis as to which muscles of lower limbs are activated by training treatment—those proximal, as a compensatory mechanism, or those distal, which are the most ischemic.

## 2. Methods

### 2.1. Subjects

The study comprised 45 patients (28 men and 17 women) aged 60–70 years (height 168.8 ± 6.8 cm, weight 78.9 ± 9.2 kg) with PAD and unilateral intermittent claudication (Fontaine stage IIa/IIb). Only patients with femoral-popliteal lesion were included. There were no patients with Leriche syndrome in this study. The diagnosis of PAD was confirmed by Doppler ultrasound and ankle/arm systolic blood pressure ratio (<0.9 at rest) [13]. They were recruited from the vascular outpatients clinic.

Patients were included in the program if their walking distance to the onset of claudication pain as measured on the treadmill (speed, 3.2 km/h; inclination, 12 degrees) was between 50 and 200 m, and claudication was stable over a 3-month period before enrollment.

Patients were excluded if they had angina pectoris, recent myocardial infarction or vascular surgery within the previous year, diabetes mellitus, cancer, kidney or liver disease, or arthritis that limited walking. Patients who were unable to walk on the treadmill at a speed of at least 3.2 km/h were also excluded. Pharmacological treatment was continued with the dosage unchanged during the study.

The participants were informed about the research protocol in detail and provided their written informed consent to participate in the study. This study was approved by Ethical Committee of the Regional Medical Chamber in Krakow (No. 30/KBL/OIL/2013, 10 April 2013). All procedures were performed in accordance with the 1964 Declaration of Helsinki and its later amendments.

### 2.2. Training Program

All the patients participated in a 12-week supervised treadmill training program. Treadmill training sessions were conducted 3 times a week during the morning hours and consisted of repetitive walking on the treadmill at a speed of 3.2 km/h and induced claudication pain within the 3rd–5th min [20]. Patients stopped exercise when level 2 on pain scale was reached [2]. After the treadmill effort, the subject stepped off the treadmill and rested until fatigue subsided from the lower limbs. Rest periods after exercises were individualized for each subject to be short and sufficient, to alleviate fatigue of the lower limbs and to undertake the next treadmill effort without pain. Both the duration and intensity of sessions were progressively increased during the program. Each week, patients were reassessed and when the patient was able to walk for 8 min or longer without reaching the onset of claudication pain, the grade of the treadmill was increased for subsequent visits to induce claudication pain within 3–5 min, as was set at the beginning of the program. For the first 2 weeks, training sessions were continued for 30 min. Afterwards, there was an increase in the session duration by 5 min per 2 weeks, up to 55 min of walking at the end of the program [20]. Training sessions were supervised and heart rate was monitored using telemetry (Polar Sport Tester M-400, Polar, Finland.

### 2.3. Methodological Approach

Measurements of the subjects’ body weight and height were taken during the first visit to the laboratory. All measurements of muscles’ bioelectrical activity were performed at baseline and after 12-weeks of treadmill training.

### 2.4. Surface Electromyography

Surface electromyography (sEMG) of the gastrocnemius lateralis (GaL), gastrocnemius medialis (GaM), tibialis anterior (TA), biceps femoris (BF), rectus femoris (RF) and gluteus medius (GM) muscles in the claudicating leg were measured according to SENIAM guidelines [21,22]. The skin was cleaned with alcohol and surface electrodes (Ag/AgCl) (Sorimex, Torun, Poland), with a 2 cm center-to-center distance, were attached along the direction of the muscle fibers.

The signals were registered with a 16-bit accuracy, at a sampling rate of 1500 Hz, using the Noraxon G2 TeleMyo 2400 unit (Noraxon USA, Inc., Scottsdale, AZ, USA). The sEMG signal was processed using MyoResearch XP software (Noraxon USA, Inc.,Scottsdale, AZ, USA) sEMG data was filtered using the built-in hardware 1st-order high-pass filter set to 10 Hz +/− 10% cut-off. The raw sEMG data were visually checked for artefacts. The sEMG signal was rectified and then the root mean squared (RMS) value was determined over a 200 msec window [22,23].

### 2.5. Maximal Voluntary Contraction (MVC)

The sEMG amplitude was normalized to maximal voluntary contraction (MVC) and expressed as %MVC [22]. The MVC for each evaluated muscle was measured during 3 s of maximal isometric effort. The measurement was repeated twice for each muscle and the highest value was determined as MVC. The MVC was measured each time before the treadmill walking effort in a stabilized position and in accordance to manual muscle testing guidelines [23].

### 2.6. Muscle Bioelectrical Activity (sEMG) Assessment during Gait

Treadmill testing was performed in the morning hours. Patients walked on the treadmill (Gait Trainer, Biodex, Shirley, NY, USA) at constant speed (3.2 km/h) and inclination angle 0% until they refused to continue because of severe claudication pain rated on a 1–5 pain scale (1—”no pain”; 2—”onset of pain”; 3—”mild pain”; 4—”moderate pain”; 5—”maximal pain”) [2]. Patients were instructed not to use handrail support during the test. The muscles’ bioelectrical activity (sEMG) was recorded continuously during the whole treadmill test. Each pain level was marked and saved on the recorded sEMG signal at the moment when it was reported by the patient. Walking time was also measured and recorded.

The following variables were calculated:

- Average mean amplitude of sEMG signal—expressed as %MVC (a decrease in the average mean amplitude after training means that the muscle responds better to the walking effort, requires less active units to perform a similar effort and this is a symptom of lower muscle fatigue);
- Mean amplitude range of the sEMG signal between minimal and maximal value measured at evaluated intervals—expressed as %MVC (a smaller mean amplitude range after training means that the muscle used less motor units to perform a similar effort and this is a symptom of lower muscle fatigue).

The following were calculated from the whole record at subsequent intervals:

- Whole measurement (entire treadmill test);
- Pain-free part of the treadmill test (from the beginning to onset of claudication pain);
- Painful part of the treadmill test (from the onset of claudication pain to the end of the test).

In order to compare changes in muscles activity after 12 weeks of training between patients with different walking times, the whole group was divided to 3 subgroups according to the walking duration they obtained after training:

- Group 1—up to 600 s (up to 10 min);
- Group 2—600–1000 s (from 10 to 16.5 min);
- Group 3—over 1000 s (over 16.5 min).

### 2.7. Statistical Analysis

Statistical analysis was carried out using STATISTICA 13.0 software. To assess the normality of variable distribution, the Shapiro–Wilk test was performed. Changes in the whole sEMG measurement over 12 weeks of the training program were analyzed by implementing ANOVA for repeated measures. One-way ANOVA was employed for the evaluation of significance in the differences of the variables between pain-free and painful intervals of the treadmill test and between groups with different walking times. Then, Tukey’s post hoc test was performed. The effect size was calculated using Cohen’s *d*. Differences were considered to be statistically significant at the level of *p* < 0.05.

## 3. Results

### 3.1. Post-Training Changes in Bioelectrical Activity of the Muscles for the Entire Treadmill Test (Total Measurement)

**Figure 1:**
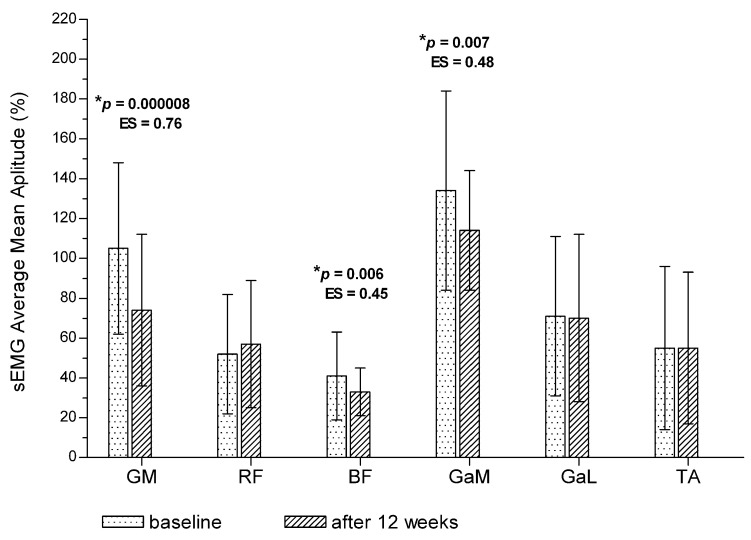
Post-training changes in average mean amplitude of sEMG for the entire treadmill test (total measurement). * *p*—*p* value between baseline and post-training value. ES—effect size (Cohen d). Values are expressed as mean ± SD.

**Figure 2:**
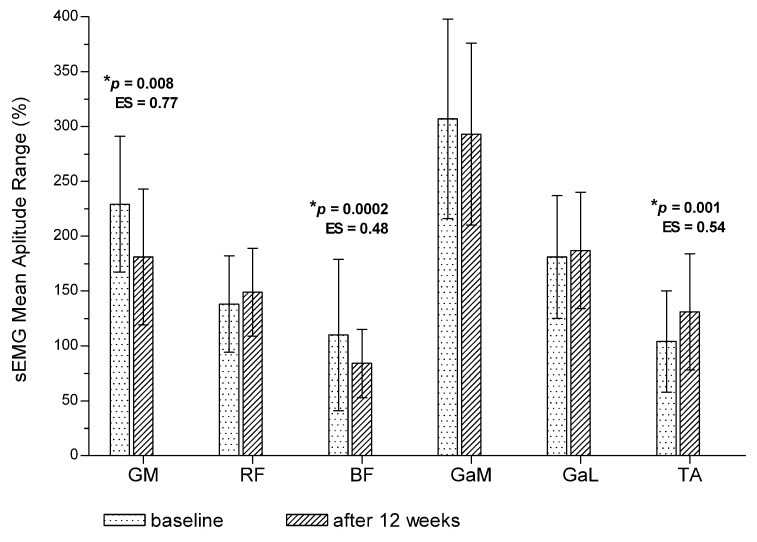
Post-training changes in mean amplitude range of the sEMG signal for the entire treadmill test (total measurement). * *p*—*p* value between baseline and post-training value. ES—effect size (Cohen d). Values are expressed as mean ± SD.

After 12 weeks of training, the average mean amplitude of the GM, BF and GaM muscles was significantly lower during the treadmill test compared with baseline (Figure 1). The mean amplitude range was significantly decreased after 12 weeks of training for the GM and BF muscles. The GaM muscle was active in a similar amplitude range as compared to baseline (Figure 2). No significant changes were noted in the remaining muscles (*p* > 0.05). On the other hand, after 12 weeks of training, the mean amplitude range of the TA muscle was significantly higher compared with baseline (Figure 2), but without significant changes in the average mean amplitude value (Figure 1).

### 3.2. Post-Training Changes in Bioelectrical Activity of the Muscles during the Pain-Free Interval of the Treadmill Test

In the pain-free condition, after 12 weeks of training, there were no significant differences in any of evaluated muscles’ bioelectrical activity compared to baseline (Table 1 and Table 2).

### 3.3. Post-Training Changes in Bioelectrical Activity of the Muscles during the Painful Interval of the Treadmill Test

When differences in muscle bioelectrical activity after training were analyzed compared with baseline for the painful walking interval, the observed changes in the Average Mean Amplitude (Table 1) and Mean Amplitude Range (Table 2) were consistent with those observed for the entire record.

### 3.4. Differences in Muscle Bioelectrical Activity between Pain-Free and Painful Intervals of the Treadmill Test

At baseline, as well as after 12 weeks of training, in none of evaluated muscles were there any significant differences between pain-free and painful intervals with regard to average mean amplitude (Table 1) or mean amplitude range (Table 2). However, the values of average mean amplitude during painful gait were higher compared with the pain-free condition, but this increase was not significant (*p* > 0.05).

### 3.5. Changes in Muscle Bioelectrical Activity among Patients with Different Walking Time

**Figure 3:**
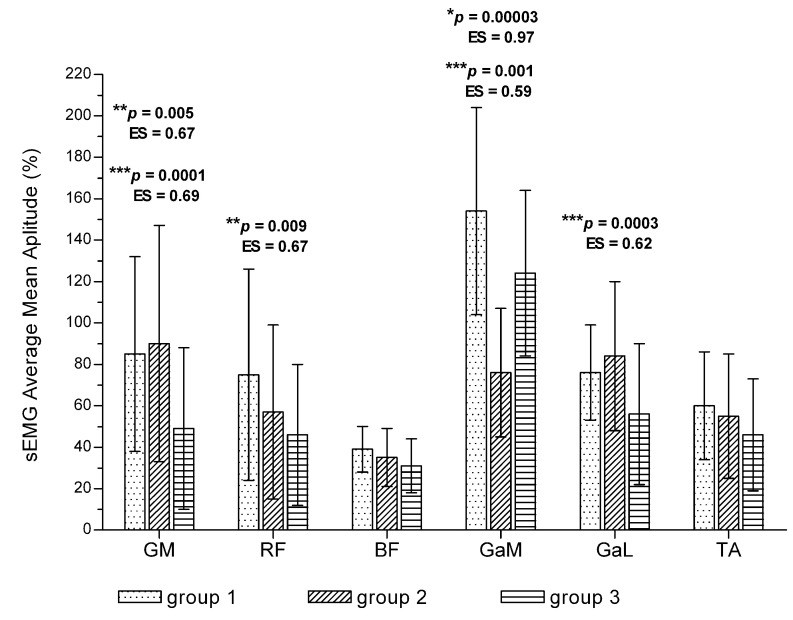
Differences in average mean amplitude of sEMG signal among patients with different walking time. *p* *—*p* value between study groups 1 and 2; *p* **—*p* value between study groups 1 and 3; *p* ***—*p* value between study groups 2 and 3. ES—effect size (Cohen d). Values are expressed as mean ± SD.

**Figure 4:**
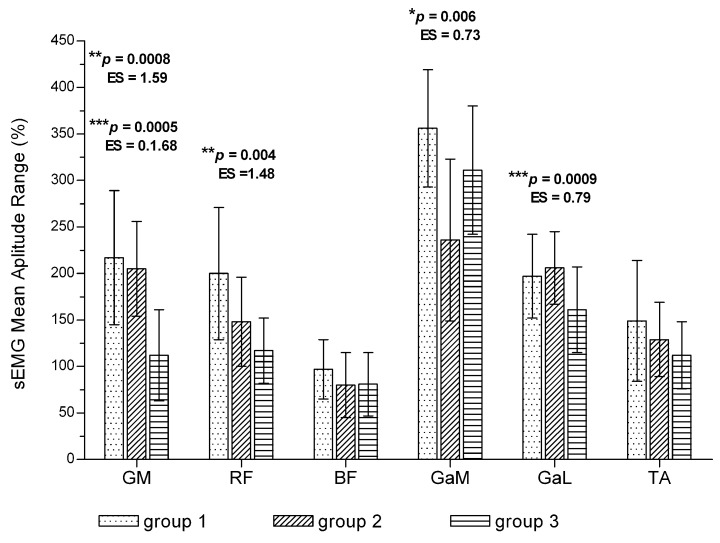
Differences in mean amplitude range of the sEMG signal among patients with different walking time. *p* *—*p* value between study groups 1 and 2; *p* **—*p* value between study groups 1 and 3; *p* ***—*p* value between study groups 2 and 3. ES—effect size (Cohen d). Values are expressed as mean ± SD.

After 12 weeks of training, the maximal walking time was significantly longer than at baseline (1066 +/− 621 s vs. 525 +/− 283 s; *p* = 0.0000; ES = 1.12). At baseline, for all the evaluated muscles, there were no significant differences between groups in average mean amplitude or mean amplitude range (*p* > 0.05). After 12 weeks of training in patients with the longest walking time (group 3), the average mean amplitude was significantly lower compared with groups with the shortest walking time in the GM, RF and GaL muscles, while in the GaM muscle, the lowest average mean amplitude was noted in group 2 (Figure 3). With regard to the GaM, RF and GaL muscles in group 3 and in GaM muscle in group 2, values observed for mean amplitude range were also significantly lower (Figure 4).

## 4. Discussion

In this study, we examined the effect of 12-week supervised treadmill training on muscle activity of the lower limbs during gait in PAD patients with intermittent claudication. We have observed that the most favorable changes in sEMG amplitude, suggesting the lowest muscle fatigue and the highest walking capacity, were in patients with the longest walking time. The observed decrease in average mean amplitude suggested that the muscles responded better to the walking effort. After 12 weeks of training, significant improvement in muscle bioelectrical activity was noted during walking for the BM, BF and GaM muscles, which means that beneficial changes were observed in both the distal and proximal muscles. However, after training, the TA muscle increased its activity, and the sEMG amplitude was significantly higher in subjects with the longest walking time, indicating that the TA muscle was more strongly engaged in the walking effort. Nonetheless, such changes did not indicate increased exercise tolerance.

In many studies, alterations in gait profile have been described concerning patients with intermittent claudication reporting that compared with the healthy population, they walk more slowly, with shorter steps, extending the stance phase while reducing the swing phase [1,4,5]. Such gait abnormalities were linked to increased energy expenditure and deterioration in gait economy [3,24,25]. However, the mechanisms underlying gait impairment in these patients are not fully understood.

Based on kinematic and kinetic data, some researchers have suggested the potential cause of these gait alterations, such as decreased muscle power in the ankle, knee and hip joints, with marked weakness regarding the posterior compartment muscles of the hip and calf [4,6]. Additionally, significant deficits in ankle plantar flexor strength have been reported, indicating that patients with PAD are unable to generate normal power for push-off, which further reduces ankle plantar flexion [7,8,9,10]. Chen et al. [11] have identified weakness in the calf muscles as a key factor underlying PAD gait adaptations. Lower range of motion in the ankle was indicated as a compensatory mechanism to minimize the duration of muscle contraction as an adaptation to painful walking [11]. Chen et al. [6] suggested that weakening of the back thigh and shin muscles affects hip joint and ankle mobility, resulting in slower gait. In our study, we have confirmed these kinematic and kinetic observations. We have noted significant improvement in the functioning of the hip extensors or plantar flexors, which resulted in improvement of walking ability.

To our knowledge, in only a few studies has muscle activity been evaluated via sEMG in patients with intermittent claudication during walking [11,26,27]. Moreover, there is lack of research in which changes concerning the bioelectrical activity of the lower limb muscles would be described after supervised treadmill training, evaluating both the proximal and distal muscles of the lower limbs. Therefore, this issue has been undertaken in this study for the first time.

According to some authors, gait abnormalities in PAD patients can be observed both during pain-free and painful conditions [1,6,24]. Other researchers have suggested that gait alterations in PAD patients were observed only after the symptoms of intermittent claudication had appeared, while prior to that moment, the gait pattern remained normal [25,28,29]. On the other hand, some authors reporting kinematic changes in gait have indicated that PAD is associated with an altered gait profile from the first step, even before the onset of claudication pain [4,11,12]. According to Schieber et al. [7], the plantar flexor muscles, even without pain, are weaker in patients with PAD compared with controls, with a further decrease in peak strength and altered muscle control strategies in the ankle with the onset of claudication pain. Interestingly, Gommans et al. [11] have noted that patients with IC showed significant changes in kinematic parameters during walking, even before the onset of claudication pain which did not intensify when this pain appeared. These changes did not coincide with differences in the sEMG muscle activity duration of the GaM and TA muscles. No significant differences have been observed between the IC patients and control subjects regarding muscular activity duration, where both groups used the GaM muscle for approximately 43% of the gait cycle. However, sEMG amplitude of both muscles did significantly increase during painful walking, but only in IC patients. These authors have suggested that pain due to chronic ischemia coincides with increased recruitment of motor units, muscle fatigue and alterations in muscle co-ordination patterns [11]. In our study, we have observed a non-significant increase in average mean amplitude for the painful condition compared with pain-free gait, baseline and after training. Moreover, in the pain-free condition, we did not observe any changes in muscle activity due to training. Without pain, muscles were active and engaged in contraction at the same level at baseline as well as after 12 weeks of training. Beneficial changes in muscle activity were observed after training compared with baseline during painful walking. The walking time was significantly longer with the lower sEMG amplitude, indicating better tolerance of this effort.

Some studies have suggested that the energy cost of locomotion is almost 40% greater in PAD patients [11,30]. Various mechanisms might be responsible, e.g., restriction of arterial blood flow to the lower limbs inducing pain [27], reduced calf muscle strength [8,9,10], or metabolic myopathy in the muscles due to exercise-induced ischemia [31,32]. The muscle phenotype is probably altered with the predominance of type II anaerobic and fatigue-prone muscle fibers, what may be associated with the exercise intolerance in these patients [27,33]. Moreover, as was reported by Askew et al. [27], calf muscle mass due to muscle fiber atrophy was reduced by 12% in the PAD group compared with control subjects. It was suggested that the loss of strength and endurance of lower limb muscles stems from histological and metabolic changes. Patients with advanced PAD suffer from atrophy of muscle fibers, which are replaced by connective tissue [34,35]. All these studies suggested changes in muscles functioning, which are present due to exercise-induced ischemia, may affect the way they react to exercise, as well as their potential for regeneration, when increased oxygen supply to working muscles is available. As was observed in our study, both the proximal and distal muscles presented potential for regeneration and increased their exercise tolerance due to 12 weeks of training.

In some studies, researchers have compared the lower limb muscle bioelectrical activity between PAD patients and healthy controls [11,18]. However, there are no studies in which changes in the bioelectrical activity of the lower limb muscles caused by the walking training program would be comprehensively examined. Only in our previous pilot study, after 12 weeks of training, did we observe improvement in bioelectrical activity of the GaL muscle [19]. In our current study, 12 weeks of supervised therapy produced a significant increase in the treadmill walking distance of patients with claudication. This increase was consistent with improvement in muscle function at the level of the ankle and hip. In our study, it has been that improvement in exercise tolerance is present in both the distal and the proximal muscles, not only the calf, as was previously suggested [7,14]. Our data remain with agreement with that provided by Schieber et al. [4], who, based on kinematic and kinetic data, reported that exercise training produced strengthening of the hip extensor as well as the ankle plantar flexor muscles, and this strengthening appears to be the main mechanism producing the improvement in walking ability among patients with claudication.

In our study, the TA muscle increased its activity. Additionally, the sEMG amplitude was significantly higher in the subjects with the longest walking time, which indicated that the TA muscle was more strongly engaged in the walking effort. Moreover, the changes in this muscle observed after training did not indicate increased walking tolerance. We have suggested that the higher TA muscle activity may possibly be the result of the greater work performed by the GaM, BF and GM muscles. It is also probable that the increase in walking ability after the training period forced the TA muscle to work longer despite the lack of beneficial sEMG amplitude changes—in other words, the stronger plantar flexors and hip extensors forced greater dorsiflexion of the foot.

This study also had some limitations. We have evaluated only one group of patients. It is probable that including a group with a different form of training, or a control group without training, could strengthen the obtained results. Moreover, due to the dynamic nature of muscle contraction during sEMG signal recording, the assessment of changes in muscle activity was based only on the signal amplitude. For more comprehensive evaluation, it would also be useful to analyze the sEMG signal frequency parameters; however, for dynamic muscle contraction, advanced wavelet analysis would be required, which—due to technical limitations—was not possible.

The obtained results may suggest that after 12 weeks of treadmill training, beneficial changes occurred in both the proximal and distal muscles. We have suggested that the gait pattern in the evaluated patients may have improved due to increased walking tolerance in the foot plantar flexors and hip extensors, which were the key limiting muscles for claudicating gait. Therefore, greater foot plantar flexion and stronger push-off, as well as greater hip extension, may be considered the main mechanisms of the observed gait pattern improvement. It may be suggested that the therapy of gait alterations in patients with PAD should be focused not only on calf muscle pump improvement, but also on proximal hip extensor strengthening. Since these muscles presented potential for improvement under the influence of physical activity, we may hypothesize that in patients with short walking distance, the use of strengthening exercises in a form other than walking may improve their functional abilities. Therefore, identifying any changes in gait, and especially in muscle activity, is important because it may help to create the most effective mode of physical therapy interventions in patients with PAD.

## Figures and Tables

**Table 1:** Comparison of average mean amplitude of sEMG signal in pain-free and in painful intervals of the treadmill test.

Outcome Measure		Pain-Free Interval	Change	*p* #	ES #	Painful Interval	Change	*p* #	ES #	*p* *	ES *
Gluteus Medius (%)	Baseline	95 ± 43	−22	0.06	0.52	116 ± 45	−43	0.0008	0.94	0.29	0.47
	Post-training	73 ± 40				73 ± 46				0.17	0.03
Rectus Femoris (%)	Baseline	50 ± 30	2	0.81	0.05	53 ± 35	6	0.38	0.16	0.78	0.09
	Post-training	52 ± 38				59 ± 37				0.51	0.18
Biceps Femoris (%)	Baseline	37 ± 21	−8	0.11	0.4	47 ± 21	−16	0.005	0.83	0.42	0.47
	Post-training	29 ± 18				31 ± 17				0.12	0.11
Gastrocnemius Medialis (%)	Baseline	134 ± 50	−21	0.19	0.46	144 ± 53	−37	0.007	0.8	0.86	0.19
	Post-training	113 ± 40				107 ± 38				0.81	0.15
Gastrocnemius Lateralis (%)	Baseline	61 ± 36	5	0.54	0.14	74 ± 33	1	0.67	0.02	0.15	0.37
	Post-training	66 ± 31				75 ± 38				0.37	0.25
Tibialis Anterior (%)	Baseline	47 ± 29	2	0.64	0.07	60 ± 24	−3	0.75	0.13	0.17	0.48
	Post-training	49 ± 28				57 ± 21				0.26	0.32

*p* #—*p* value between baseline and post-training value. *p* *—*p* value between pain-free and painful intervals. ES #—effect size (Cohen d) between baseline and post-training value. ES *—effect size (Cohen d) between pain-free and painful intervals. Average mean amplitude of sEMG signal was expressed as %MVC (Mean ± SD). Change (post-training to baseline) was expressed as %MVC—negative value means that the muscle responds better and positive value means that the muscle responds worse to the walking effort.

**Table 2:** Comparison of mean amplitude range of the sEMG signal between minimal and maximal value in pain-free and in painful intervals of the treadmill test.

Outcome Measure		Pain-Free Interval	*p* #	ES #	Painful Interval	*p* #	ES #	*p* *	ES *
Gluteus Medius (%)	Baseline	246 ± 73	0.12	0.69	257 ± 55	0.008	1.74	0.63	0.17
	Post-training	194 ± 77			170 ± 44			0.37	0.38
Rectus Femoris (%)	Baseline	149 ± 52	0.34	0.09	137 ± 35	0.32	0.11	0.72	0.27
	Post-training	154 ± 55			141 ± 32			0.23	0.28
Biceps Femoris (%)	Baseline	111 ± 69	0.07	0.46	123 ± 54	0.003	0.79	0.92	0.19
	Post-training	86 ± 32			86 ± 37			0.96	0.03
Gastrocnemius Medialis (%)	Baseline	319 ± 81	0.69	0.07	305 ± 90	0.57	0.19	0.73	0.16
	Post-training	313 ± 83			288 ± 85			0.3	0.29
Gastrocnemius Lateralis (%)	Baseline	183 ± 56	0.38	0.25	181 ± 50	0.45	0.09	0.9	0.03
	Post-training	197 ± 53			176 ± 55			0.55	0.38
Tibialis Anterior (%)	Baseline	111 ± 49	0.12	0.43	104 ± 43	0.01	0.81	0.59	0.15
	Post-training	134 ± 57			144 ± 55			0.92	0.17

*p* #—*p* value between baseline and post-training value. *p* *—*p* value between pain-free and painful intervals. ES #—effect size (Cohen d) between baseline and post-training value. ES *—effect size (Cohen d) between pain-free and painful intervals.. Mean amplitude range of the sEMG signal of sEMG signal was expressed as %MVC (Mean ± SD).

## Data Availability

All data generated or analyzed during this study are included in this article. The datasets used during the current study are available from the corresponding author on reasonable request.
